# Prognostic Factors in Lung Adenocarcinoma with Bone Metastasis Treated with EGFR-TKIs

**DOI:** 10.3390/medicina57090967

**Published:** 2021-09-15

**Authors:** Tzu-Hsuan Chiu, Chun-Yu Lin, Meng-Heng Hsieh, Shu-Min Lin, Yueh-Fu Fang

**Affiliations:** 1 Department of Thoracic Medicine, Chang Gung Memorial Hospital at Linkou, Taoyuan 33305, Taiwan; aglaonema@cgmh.org.tw (T.-H.C.); pitiful1984@gmail.com (C.-Y.L.); mengheng@cgmh.org.tw (M.-H.H.); smlin100@gmail.com (S.-M.L.); 2 Department of Pulmonary and Critical Care, Saint Paul’s Hospital, Taoyuan 33069, Taiwan

**Keywords:** survival, denosumab, EGFR-TKIs, afatinib, bone metastasis, lung cancer

## Abstract

*Background and Objectives:* Patients who have advanced lung cancer and bone metastasis (BM) often suffer from skeletal-related events (SREs) that lead to poor quality of life and poor prognosis. Our study aimed to investigate the prognostic factors in patients with BM from epidermal growth factor receptor (EGFR) mutation-positive lung adenocarcinoma. *Materials and Methods:* This retrospective study included 77 lung adenocarcinoma patients with synchronous BM. These patients had first-line EGFR tyrosine kinase inhibitors (EGFR-TKIs) between January 2017 and December 2019. Among them, 42 patients were treated with 120 mg of subcutaneous denosumab monthly. We investigated their baseline characteristics, cancer management, SREs, progression-free survival (PFS), and overall survival (OS). *Results:* The PFS in the patients treated with or without denosumab were 10.1 vs. 12.5 months (*p* = 0.971). The median OS was 26.9 vs. 29.5 months (*p* = 0.967) in no denosumab and denosumab groups, respectively. Univariate analyses showed benefit of afatinib in PFS and good performance status in OS. *Conclusion*: Those patients that took afatinib as first-line EGFR-TKIs had significantly longer PFS than those treated with other TKIs. Denosumab had no prognostic effect on PFS or OS.

## 1. Introduction

Bone metastasis (BM) is common in patients with non-small-cell lung carcinoma (NSCLC), often causing pain and poor quality of life [1]. BM also shows poor survival in patients with lung cancer, especially after the first skeletal-related event (SRE) has developed [1,2]. Bisphosphonate therapy and palliative radiotherapy may decrease SREs and prolong overall survival [1,3]. Denosumab, a humanized inhibitor of nuclear factor κB ligand (RANKL), is used to prevent or delay SREs in patients with advanced cancer metastatic of the bone [4]. Moreover, in the subgroup analysis of a phase III study, NSCLC patients treated with denosumab showed improved overall survival (OS) compared to those treated with zoledronic acid (ZA) [5]. Udagawa et al. [6] also demonstrated that denosumab therapy had better OS in patients with BMs from non-squamous NSCLC compared to the ZA group. Peters et al. reported there was no OS improvement with denosumab in lung cancer patients treated with first-line chemotherapy in the phase III SPLENDOUR trial [7].

Afatinib, a second-generation irreversible EGFR-TKI, was approved as the first-line treatment for EGFR mutation-positive NSCLC in 2013 [8]. Ihn et al. [9] reported that afatinib attenuated osteoclast activity and function by downregulating the RANK signaling pathway. As mentioned in previous studies [5,6], denosumab may have a survival benefit in patients with lung cancer. However, few studies have focused on the effect of denosumab in patients with BM from advanced NSCLC treated with first-line EGFR-TKIs. Therefore, this study aimed to investigate the prognostic factors in patients with BM from EGFR mutation-positive lung adenocarcinoma, including the effect of denosumab.

## 2. Methods

### 2.1. Patients

This retrospective study included adult patients who had lung adenocarcinoma with synchronous BM receiving first-line EGFR-TKIs treatment (gefitinib, erlotinib, and afatinib) between January 2017 and December 2019 at the Linkou Medical Center of Chang Gung Memorial Hospital. The diagnosis of BM was based on CT imaging findings of bone scintigraphy or 18FDG-PET. Depending on the treatment for the BM concurrently with the first-line EGFR-TKI therapy, the patients were divided into subgroups: no denosumab and denosumab groups. Patients in the denosumab group were treated with 120 mg of subcutaneous denosumab monthly within 1 month after diagnosis of BM. The patients did not receive zoledronic acid or other agents for their bone metastasis. Data on patient characteristics were collected, including age, sex, EGFR mutation status, and Eastern Cooperative Oncology Group (ECOG) score. SREs were defined as pathologic fractures, spinal cord compression, radiation, or surgery to bone. The objective tumor response was assessed according to the Response Evaluation Criteria in Solid Tumors, version 1.1 [10]. Tumor assessments were performed every three months in almost all patients. OS was defined as the interval between the initiation of first-line EGFR-TKIs and the date of death from any cause. Progression free survival (PFS) was calculated from the date of initiation of the first EGFR-TKIs until the date of detection of disease progression or date of death from any cause. This study was approved by the institutional review board of Chang Gung Memorial Hospital (no. 201901736B0). As this was a retrospective study and no modification in the management of patients was required, the need for informed consent was waived.

### 2.2. Statistical Analysis

A chi-square test was used to determine the statistical significance of differences among groups. A Kaplan-Meier survival analysis was performed to analyze PFS and OS. The differences in OS and PFS were compared using the log-rank test. Statistical significance was set at *p* < 0.05. Survival was assessed until 31 December 2019. Statistical analyses were performed using GraphPad Prism statistical software, version 6 (GraphPad Software, La Jolla, CA, USA) and IBM SPSS Statistics 20 for Mac (SPSS, Chicago, IL, USA).

## 3. Results

### 3.1. Patients Characteristics

The characteristics of the 77 patients are summarized in Table 1. Forty-two patients were concurrently treated with denosumab. Age, gender, and ECOG score were similar between groups. All patients had EGFR mutations. The most frequent EGFR mutations were the exon 21-point mutation L858R (39 patients, 50.6%) and exon 19 deletion (33 patients, 42.9%). Prior SREs were comparable in both the no denosumab and denosumab groups (29% vs. 36%, *p* = 0.626). Around half the patients underwent first-line afatinib therapy. 

### 3.2. Analysis of PFS and OS

**Figure 1:**
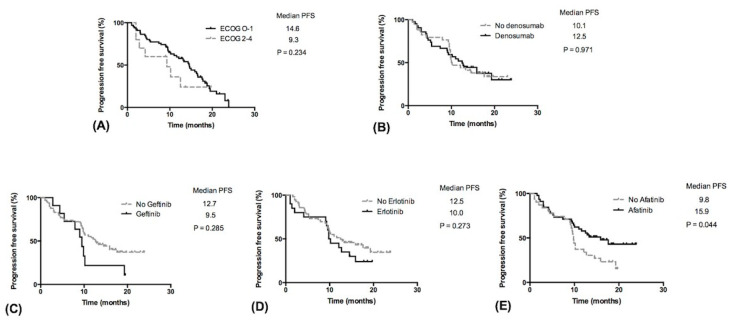
Progression free survival (PFS) in patients with lung adenocarcinoma and bone metastasis. (**A**) PFS in relation to ECOG score, (**B**) PFS in relation to denosumab treatment, (**C**–**E**) PFS in relation to first-line EGFR-TKIs. Abbreviations: PFS, progression-free survival; ECOG, Eastern Cooperative Oncology Group.

The median follow-up time was 35.2 months. Twenty-two (63%) patients had progressive disease in the no denosumab group and 28 (80%) in the denosumab group; *p* = 1.0. The median PFS was 10.1 months in the no denosumab group and was 12.5 months in the denosumab group (*p* = 0.971) (Figure 1B and Table 2). Age, ECOG score 0–1, and SRE were not related to PFS (Table 2). Among treatments with EGFR-TKIs, patients managed with or without gefitinib and erlotinib had comparable PFS (Figure 1C,D, Table 2). The median PFS in the afatinib group was significantly longer than that in patients treated with other TKIs (15.9 vs. 9.8 months, *p* = 0.044, Figure 1E). The risk of progressive disease was reduced in the afatinib group with a hazard ratio (HR) of 0.57 (95% CI: 0.30–0.98, Table 2). 

**Figure 2:**
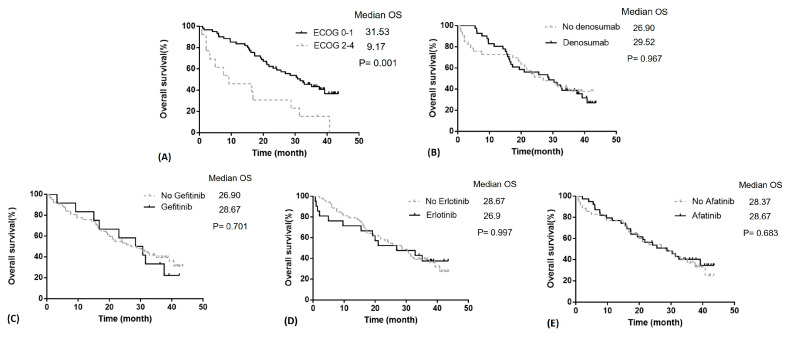
Overall survival (OS) in patients with lung adenocarcinoma and bone metastasis. (**A**) OS in relation to ECOG score, (**B**) OS in relation to denosumab treatment, (**C**–**E**) OS in relation to first-line EGFR-TKIs. Abbreviations: OS, overall survival; ECOG, Eastern Cooperative Oncology Group.

The median OS was 26.9 vs. 29.5 months (*p* = 0.967) in no denosumab and denosumab groups (Figure 2B). First-line EGFR-TKIs therapy with gefitinib, erlotinib, or afatinib had similar OS rates (Table 3 and Figure 2C–E). Age and SRE were not related to OS (Table 3). Patients with an ECOG score of 0–1 showed significantly better OS compared to those with an ECOG score of 2–4 (median OS: 31.5 in ECOG 0–1 vs. 9.17 months in ECOG 2–4, *p* = 0.001, Figure 2A). A better performance status, ECOG score 0–1, demonstrated a lower risk of mortality (HR = 0.36, 95% CI: 0.09–0.55, *p* = 0.001, Table 3).

## 4. Discussion

In our 77 patients, with a median follow-up duration of 17.2 months, denosumab had no significant survival implication. Afatinib seemed to have a more potent effect on PFS among patients with BM from lung adenocarcinoma. The ECOG status remains the most important predictive factor for OS. 

The incidence of BM in NSCLC is 30–40%, and 60% of these patients present with BM at the time of diagnosis [11]. The presence of BMs seems to represent a negative prognostic factor for patients with NSCLC [3]. Denosumab, as a bone-targeted agent, is beneficial in the prevention of SREs and in the reduction of bone pain [12,13]. Recently, Ihn et al. [8] reported that afatinib, an irreversible, second-generation EGFR-TKI, suppresses RANKL-induced osteoclast differentiation, downregulates the expression of osteoclast-specific markers, and attenuates bone resorption activity. In the current study, there was no difference in SREs between the denosumab and no denosumab groups. The univariant analysis showed better PFS in patients that had first-line afatinib in EGFR-mutant lung cancer patients with synchronous BM. A total of 49% patients in the no-denosumab group and 67% of patients in the denosumab group had first-line afatinib, which improved the PFS and was probably beneficial in the control of BM and SREs. We may need a prospective trial to study the combined effect of denosumab and afatinib.

In addition to reducing SREs, bisphosphonates and denosumab were also reported to improve OS and PFS in a systematic review [12]. A post hoc analysis conducted by Scagliotti et al. [5] included 811 adult patients with lung cancer and NSCLC. In patients with lung cancer (all types), denosumab prolonged median OS by 1.2 months compared with that by ZA (8.9 vs. 7.7 months; *p* = 0.01). In the subgroup of patients with NSCLC, denosumab prolonged median OS by 1.5 months compared with that by ZA (9.5 months vs. 8.0 months, *p* = 0.01). In the subgroup analysis, patients with squamous cell carcinoma and denosumab had an OS that was 2.2 months longer than the ZA group (8.6 months vs. 6.4 months; *p* = 0.035). However, in patients with adenocarcinoma, although the median OS was longer in the denosumab group (9.6 months vs. 8.2 months; *p* = 0.075), this difference was not statistically significant. In another retrospective study, Udagawa et al. [6] reviewed 149 patients with BM from non-squamous NSCLC and divided them into three groups: 52 received denosumab (Dmab group), 51 received zoledronic acid (ZA group), and 46 received no treatment (No-Tx group) for the BM [6]. The median OS in the Dmab group, ZA group, and No-Tx group was 21.4 months, 12.7 months, and 10.5 months, respectively. They demonstrated that denosumab treatment was significantly associated with a more favorable survival (HR = 0.5; 95% CI 0.332–0.741; *p* < 0.01). However, in the above investigations, most patients had received prior treatment, including systemic chemotherapeutic agents, and there were no data on EGFR mutations or treatment with EGFR-TKIs that strongly influenced the survival of patients with non-squamous NSCLC. Compared with gefitinib, afatinib seems to be associated with longer PFS as a first-line treatment for EGFR mutant patients [14,15]. However, in LUX-Lung 7, there was no significant difference in OS with afatinib versus gefitinib [16]. As Ihn et al. [9] reported, afatinib may suppress osteoclastogenesis by downregulating RANK signaling pathways and reducing osteolysis after BM. The implication of afatinib in survival among lung cancer patients with BMs remains unknown. In contrast to the findings of Scagliotti et al. [5] and Udagawa et al. [5,6] (Scagliotti et al., 2012; Udagawa et al., 2017) [5,6], in the current study, we found that denosumab had no prognostic effects in patients with lung adenocarcinoma and BM who were treated with first-line EGFR-TKIs. Moreover, although the OS was not different among the three EGFR-TKIs, the first-line treatment with afatinib significantly prolonged the PFS (median PFS 15.9 vs. 9.8 months, Figure 1E). While Kuan et al. [13] and Park et al. [14] found that afatinib was associated with longer PFS in first-line treatment, they did not perform further subgroup analysis among patients with BM. In the current analysis, we suggest that the outcome benefits of afatinib may be attributed to the effect of controlling BM via concurrent inhibition of RANK signaling pathways.

Morbidity associated with the skeleton is an important issue in patients with BM from NSCLC and contributes to patients’ performance status (PS). Improvement in systemic therapy, including immunotherapy and TKIs, has prolonged OS and the incidence of SREs has also increased [3]. Delea et al. [17] conducted a large prospective study and found that SREs not only lead to deterioration of PS, but also to increased economic costs. In our study, we demonstrated that PS, as the ECOG score, was the only prognostic factor for OS. Thus, the best therapeutic approach should be the prevention of SREs and the maintenance of good ECOG PS. 

Our study has several inherent limitations. First, the retrospective non-randomized study was conducted at a single institution, and the sample size was relatively small. Second, we did not analyze potential biomarkers, such as RANKL, RANK, and biomarkers of bone turnover. Third, there were more patients treated with afatinib in the denosumab group in our retrospective study.

## 5. Conclusions

In summary, our study demonstrated that denosumab had no prognostic effects in patients with lung adenocarcinoma and BM who were treated with first-line EGFR-TKIs. Afatinib had a better PFS among these patients. A better PS with an ECOG score of 0–1 was the only prognostic factor for OS. Further prospective studies are warranted, with a larger number of patients, which focus on lung adenocarcinoma with BM and treatment with EGFR-TKIs. 

## Figures and Tables

**Table 1:** Patient characteristics.

	Denosumab N = 42 (%)	No Denosumab N = 35 (%)	All N = 77 (%)	*p*-Value
Sex, Male	13 (31)	11 (31)	24 (31)	1.0
Age, years	64.5 ± 1.5	63.6 ± 1.9	64.1	0.704
EGFR mutation				0.2936
Exon 21 L858R	25 (60)	14 (40)	39 (51)	
Exon 19 deletion	13 (31)	20 (57)	33 (43)	
Rare mutation	4 (10)	1(3)	5 (6)	
ECOG				1.0
0–1	34 (81)	28 (80)	62 (81)	
Skeletal-related events	15 (36)	10 (29)	25 (32)	0.626
Radiation or surgery to bone	12 (29)	7 (20)	19 (25)	0.436
Spinal cord compression	4 (10)	2 (6)	6 (8)	0.683
Pathological fracture	5 (12)	5 (14)	10 (18)	1.0
EGFR-TKIs				0.276
Gefitinib	6 (14)	5 (14)	11 (14)	
Erlotinib	8 (19)	13 (37)	21 (27)	
Afatinib	28 (67)	17 (49)	45 (58)	

**Table 2:** Univariate analysis for progression free survival.

	Univariate Analysis	
	HR (95% CI)	*p*-Value
Age ≥ 65 years	1.04 (0.59–1.83)	0.900
ECOG score 0–1	0.62 (0.21–1.45)	0.234
Skeletal-related events	1.30 (0.71–2.44)	0.388
Denosumab	0.99 (0.56–1.75)	0.971
Gefitinib	1.75 (0.84–4.87)	0.285
Erlotinib	1.40 (0.75–2.79)	0.273
Afatinib	0.57 (0.30–0.98)	0.044

**Table 3:** Univariate analysis for overall survival.

	Univariate Analysis	
	HR (95% CI)	*p*-Value
Age ≥ 65 years	1.41 (0.81–2.52)	0.229
ECOG score 0–1	0.36 (0.09–0.55)	0.001
Skeletal-related events	1.03 (0.58–1.84)	0.926
Denosumab	1.01 (0.57–1.80)	0.967
Gefitinib	1.15 (0.54–2.48)	0.701
Erlotinib	1.00 (0.53–1.89)	0.997
Afatinib	0.89 (0.50–1.57)	0.683

## Data Availability

The study data are available on reasonable request from the corresponding author.
